# Tuberculosis Treatment Outcome in Patients with TB-HIV Coinfection in Kuala Lumpur, Malaysia

**DOI:** 10.1155/2021/9923378

**Published:** 2021-05-29

**Authors:** Diana Safraa Selimin, Aniza Ismail, Norfazilah Ahmad, Rohani Ismail, Nurul Farhana Mohd Azman, Amaleena Azman

**Affiliations:** ^1^Department of Community Health, Faculty of Medicine, Universiti Kebangsaan Malaysia Medical Centre, 56000 Kuala Lumpur, Malaysia; ^2^The Federal Territory of Kuala Lumpur and Putrajaya Health Department, 56000 Kuala Lumpur, Malaysia

## Abstract

**Background:**

Tuberculosis (TB) is a serious health threat to people living with human immunodeficiency virus (HIV). This study aimed to identify the characteristics, unsuccessful TB treatment rate, and determinants of unsuccessful TB treatment outcome among patients with TB-HIV coinfection in Kuala Lumpur.

**Methods:**

This was a cross-sectional study. The data of all patients with TB-HIV in the federal territory of Kuala Lumpur from 2013 to 2017 were collected and reviewed. The data were retrieved from the national database (TB Information System) at the Kuala Lumpur Health Department from 1 March 2018 to 31 May 2018.

**Results:**

Out of 235 randomly selected patients with TB-HIV, TB treatment outcome was successful in 57.9% (cured and completed treatment) and unsuccessful in 42.1% (died, failed, or lost to follow-up). Patients who did not receive DOTS (directly observed treatment, short course) (adjusted odds ratio: 21.71; 95% confidence interval: 5.36–87.94) and those who received shorter treatment duration of <6 months (aOR: 34.54; 95% CI: 5.97–199.93) had higher odds for unsuccessful TB treatment outcome.

**Conclusions:**

Nearly half of the patients with TB-HIV had unsuccessful TB treatment outcome. Therefore, it is important to ensure that such patients receive DOTS and continuous TB treatment of >6 months. It is crucial to strengthen and widen the coverage of DOTS, especially among high-risk groups, in healthcare settings. Strict follow-up by healthcare providers is needed for patients with TB-HIV to gain treatment adherence and for better rates of successful TB treatment.

## 1. Introduction

It is undeniable that tuberculosis (TB) and human immunodeficiency virus (HIV) coinfection poses a major public health threat worldwide [1, 2]. Worldwide, it has been estimated that more than one-third of people living with HIV (PLHIV) are infected with TB [2, 3]. Out of 10 million people infected with TB in 2019, 8.2% was PLHIV [4]. An estimated 70.0% of PLHIV are from sub-Saharan African countries [5]. Despite the Southeast Asian Region (SEAR) experiencing 34% decreased TB incidence among PLHIV within an 11-year period until 2013, high TB-HIV disease burden was still observed in Indonesia, Myanmar, Thailand, India, and Nepal [6]. In order to reduce the incidence of TB disease among PLHIV, 32% PLHIV enrolled for HIV care in SEAR countries received TB preventive treatment in 2019 [7]. In year 2020, 208000 TB deaths were observed among PLHIV which was a reduction from 678000 in year 2000 [4].

The prevalence of TB-HIV coinfection in Malaysia was 12.6% on 2010 [8] and the latest in 2019 was 5.9% [9]. Eventhough TB prevalence keeps increasing in Malaysia since year 2000, the prevalence of TB-HIV coinfection has been observed to maintain less than 6% since year 2014. In Malaysia, management of coinfection among PLHIV in Malaysia incorporates TB screening among PLHIV and HIV screening among TB patients since 1997. Ministry of Health of Malaysia has started TB screening in settings such as prisons and drug rehabilitation centres since year 2013. Between year 2015 and 2017, it was shown that active TB occurrence among newly enrolled PLHIV decreased from 9% to 4.7%. In year 2010, in an effort to reduce morbidity and mortality of TB/HIV coinfection, Malaysia started isoniazid prophylaxis. The coverage for prophylaxis among patients with TB-HIV was 70%–79% between 2015 and 2017 [10].

TB is known as the most common opportunistic infection among PLHIV; treatment management is complicated and TB is the main culprit in most HIV deaths [2, 3, 11]. The World Health Organization (WHO) has reported that around 400000 PLHIV deaths are due to TB infection [3]. PLHIV have a higher risk for latent TB infection to progress to active TB. As a result of active TB, the immune system of PLHIV is suppressed further as the viral load increases and CD4 levels decrease. Managing TB-HIV coinfection presents enormous challenges to physicians [12].

Patients with TB-HIV without antiretroviral therapy (ART) tend to have poorer TB outcome compared to those who are on ART [8, 13, 14]. ART should not been delayed especially among MDR-TB patients with HIV coinfection [15]. Study showed that the rate of unsuccessful MDR-TB treatment is proportionately increasing with the increasing frequency of missed clinic visits [16]. TB infection with late presentation and HIV diagnosis are further risk factors for unsuccessful TB treatment outcome among patients with TB-HIV [17–19]. Other than that, patients with positive sputum culture upon TB diagnosis are at higher risk for unsuccessful TB treatment outcome [20].

The Directly Observed Treatment, Short Course (DOTS) strategy has been introduced by the WHO since year 1994. It is an effort that required full support and commitment from every government for its implementation. TB case detection is done via passive case findings. This strategy highlighted a standardization of short-course TB treatment to all confirmed TB sputum smear-positive cases which can be given by the healthcare providers or family members to ensure compliance of TB treatment. Other than that, this strategy also ensures all essential anti-TB drugs are supplied regularly. Programme supervision and evaluation are also established as part of its monitoring system [21].

The Regional Strategic Plan towards Ending TB in the SEAR 2016–2020 was implemented to achieve successful TB elimination in the region by 2035. Hence, TB and HIV programmes need to be strengthened and aimed towards successful implementation by understanding the characteristics of patients with TB-HIV with successful or unsuccessful treatment outcomes [6]. However, studies, especially local studies, for determining the characteristics of such patients, including their clinical status [22], are scarce to date.

The objective of the present study was to identify the characteristics of patients with TB-HIV, describe the TB treatment outcome, and identify the associated factors for unsuccessful TB treatment outcome in such patients in Kuala Lumpur, Malaysia.

## 2. Materials and Methods

### 2.1. Study Population and Sampling

This was a cross-sectional study involving patients with TB-HIV coinfection in Kuala Lumpur, Malaysia. The sample population was patients with TB who were notified and registered with the National Registry for the TB database, i.e., the National Tuberculosis Information System (TBIS), by the Kuala Lumpur Federal Territory Health Department. Patients with TB-HIV were included via simple random sampling from the patient name list, and TBIS served as the sampling frame. Sample size was calculated based on the formula by Kish [23] and in reference to a previous local study [8]. After considering 20% missing data, a minimum of 235 patients with TB-HIV was included in this study.

**Figure 1:**
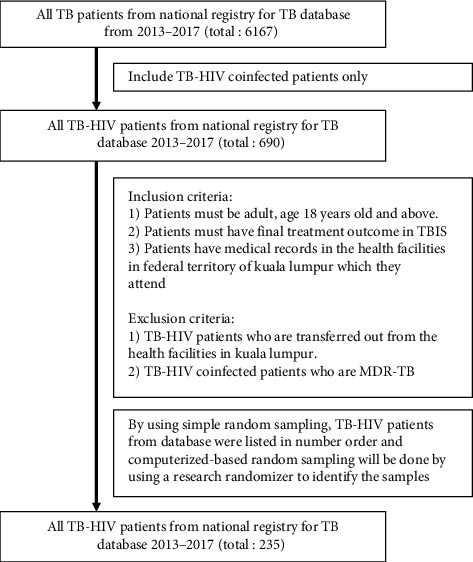
The recruitment of TB-HIV patients for this study.

TB-HIV patients' recruitment for this study was simplified in the flowchart (Figure 1).

### 2.2. Data Collection

The TB records of all patients with TB-HIV in Kuala Lumpur from 2013 to 2017 were retrieved, reviewed, and collected from the TBIS database at the Kuala Lumpur Federal Territory Health Department.

TBIS database is a health database, under Ministry of Health of Malaysia. Data in this database were collected nationwide, for the purpose of monitoring and surveillance TB disease in Malaysia. HIV department and TB department were monitored under the Infectious Disease Control Division, Ministry of Health of Malaysia. Both departments work together to accomplish better outcome for TB-HIV patients in Malaysia. However, datasets used for analysis in this study are not publicly available due to some concern, in which it contained health information which could compromise the privacy of research participants. Details on TBIS also have been cited in few other studies related to TB in Malaysia [24–26].

### 2.3. Outcome Variables

The study outcome was successful or unsuccessful TB treatment. Unsuccessful TB treatment was defined as death (for any reason during the treatment course), treatment failure (positive sputum smear at 5 months or later during treatment), or lost to follow-up (interrupted treatment for >2 consecutive months).

Successful TB treatment was defined as when a patient was cured (previously smear-positive patients that were smear-negative in the final month of treatment and at least once on a previous occasion) and had completed treatment (patient had completed treatment but did not meet the criteria to be classified either as cure or failure).

### 2.4. Independent Variables

The sociodemographic characteristics included in the study were age, sex, citizenship status, ethnicity, marital status, and place of residence. Age was counted starting from the date of birth until the TB notification date. Sex was classified as male or female; citizenship status was classified as Malaysian or non-Malaysian. Ethnicity was considered race inherited from parents, e.g., Malay, Chinese, Indian, or others. For place of residence, all flats and slums were considered low-cost residential areas. Apartments, condominiums, terrace houses, and bungalows were considered medium- or high-cost residential areas; the place of residence of patients who were homeless or who were institutionalized, i.e., in detention centres or prisons, was classified as “others.”

The socioeconomic characteristics included in the study were formal education, employment status (employed or unemployed), and household income. Any formal education, regardless of duration, was classified as “yes,” and no formal education was classified as “no.” Patients who were employed, including self-employment, were categorized as “employed,” and patients who did not work were categorized as “unemployed.” Household income was considered low when it was under 3000 Malaysian ringgit (<MYR3000) and was considered high when it was ≥MYR3000.

The clinical characteristics retrieved were diabetes mellitus (DM) status, smoking status, Bacille Calmette-Guérin (BCG) scar status, ART status, TB type, TB case category, chest X-ray (CXR) presentation upon diagnosis, sputum smear upon diagnosis, sputum culture upon diagnosis, directly observed treatment, short course (DOTS) status, and duration of TB treatment. For DM status, patients with underlying DM were categorized as “yes,” and those without DM were categorized as “no.” For smoking status, smokers were classified as “yes,” and nonsmokers were classified as “no.” Patients with a BCG scar on any part of the body, as it varies by country, were categorized as “present,” and those without a BCG scar were categorized as “absent.” Patients who received ART were categorized as “yes,” and those who did not receive ART were categorized as “no.” In this study, TB type was divided into two categories based on the anatomical site of disease: pulmonary and extrapulmonary. Pulmonary tuberculosis was defined as any bacteriologically confirmed or clinically diagnosed case of TB involving the lung parenchyma or the tracheobronchial tree, which includes military TB. Any bacteriologically confirmed or clinically diagnosed case of TB involving organs other than the lungs was considered as extrapulmonary tuberculosis. Cases with both pulmonary and extrapulmonary TB such as disseminated TB were classified as pulmonary TB [27]. TB case categories were divided into new cases, relapse and return after lost to follow-up. New cases were defined as all new TB cases notified among HIV patients. Relapse indicates patient whose most recent treatment outcome was “cured” or “treatment completed” and subsequently diagnosed with positive TB by sputum smear microscopy or culture. Meanwhile, return after lost to follow-up was defined as stopping TB treatment before the completion of treatment. As it is possible for a patient to enter several TB case categories, we only take their latest outcome for the purpose of this study, which means there will be no overlapped outcome for one patient. This goes the same for the patient's treatment, in which we look at their latest treatment and duration of treatment they received.

CXR presentation upon diagnosis was categorized according to how severe the lesion appeared on the X-ray film: “no or minimal lesion” if CXR showed no or few lesions, “advanced lesion” if CXR showed extensive lesions or miliary appearance, and “not performed” if CXR was not performed upon diagnosis. Sputum smear and sputum culture were both categorized as “positive” if the microscopic examination suggestive confirmed the presence of AFB in the smear or culture, “negative” if the microscopic examination did not have evidence of the presence of AFB in the smear or culture, and “not performed” if no sputum smear or culture were performed upon diagnosis. To ensure compliance of TB treatment, DOTS was applied by using healthcare providers and family members. DOTS status was “yes” if the patient received DOTS and “no” if the patient did not receive DOTS. TB treatment duration was divided into three categories: <6 months, 6–12 months, and >12 months.

### 2.5. Statistical Analysis

Data were analysed using SPSS 22. For descriptive analysis, continuous data were reported as the mean and standard deviation (SD) as the data were distributed normally. Categorical data are reported as the frequency (*n*) and percentage (%). The association between the independent variables and outcome variable (unsuccessful TB treatment outcome) was determined using simple logistic regression (SLR). Multivariable analysis was conducted using multiple logistic regression (MLR) analysis to obtain the adjusted odds ratio (aOR) and 95% confidence interval (CI) and to control for possible confounders. The significance level was set at *p* < 0.05.

## 3. Results and Discussion

### 3.1. Results

From 2013 to 2017, there were total of 690 patients with TB-HIV. Of these, 235 patients were included in the study. Patients who were transferred out, aged <18 years and with multidrug resistance TB (MDR-TB), were excluded from the study. The mean patient age was 39.49 (SD 9.35) years. Most of the patients were Malaysians (89.8%), male (85.5%), Malay (48.9%), and lived in medium- or high-cost residences (62.1%) (Table 1).

Most of the patients had received formal education (79.6%), were unemployed (51.1%), and had low household income, i.e., <MYR3000 (88.5%) (Table 1).

The majority of patients was classified as new TB cases (84.7%); 74% was classified as pulmonary TB. Most patients did not receive ART (60.9%), were nondiabetic (96.2%), nonsmokers (51.5%), and had BCG scars (90.6%). Upon diagnosis, the majority had no or minimal lesion on CXR (67.7%), 50.6% had negative sputum smear, and 63.4% had negative sputum culture. Most patients were under DOTS (66%); 48.1% had 6–12 months TB treatment. One hundred and thirty-six patients (57.9%) had successful TB treatment outcome, and 99 patients (42.1%) had unsuccessful TB treatment outcome (Table 2).

SLR showed that a few significant factors were associated with TB treatment outcome, namely, citizenship status, ethnicity, formal education received, employment status, household income, BCG scar, ART, CXR upon diagnosis, sputum culture upon diagnosis, DOTS status, and TB treatment duration (Table 3). After adjusting for other factors, the determinants for TB treatment outcome were determined using binary logistic regression.  Table 4 provides the final model. Patients who did not receive DOTS had 22 times higher odds of having unsuccessful TB treatment outcome (aOR: 21.71, 95% CI: 5.36–87.94, *p* ≤ 0.001). Patients with shorter TB treatment duration, i.e., <6 months, had 35 times higher odds of having unsuccessful TB treatment outcome (aOR: 34.54, 95% CI: 5.97–199.93, *p* ≤ 0.001). This model had no multicollinearity and was stable (variance inflation factor [VIF] <10), and there was no interaction problem.

## 4. Discussion

In the present study, TB treatment outcome in patients with TB-HIV was closely associated with that of a study performed in 2010 in the Klang Valley, Malaysia, that reported 53.4% successful treatment outcome and 46.6% unsuccessful treatment outcome [8]. However, in the district of Kota Baharu, Malaysia, 93% of patients with TB-HIV had successful treatment outcome [28]. By comparison, other studies conducted in South Africa also showed better TB treatment outcome in patients with TB-HIV [29]. Therefore, these differences must be due to multifactorial aspects such as diverse outlook on sociodemographic structure and service provision settings.

In this study, the determinant factors for unsuccessful TB treatment outcome were as follows: not receiving DOTS and TB treatment duration of <6 months. Our findings are supported by other studies that reported that DOTS can improve the cure rate [11, 30]. Study carried out in Malaysia showed that the longer waiting time spent by patients at the DOTS centre, there will be higher the risk of treatment interruption. Patients felt uneasy as they require regular medical leave from their jobs, thus affecting their income [31]. Longer waiting time also is a proxy towards unsatisfaction of the healthcare system delivery, which results in high drop out from DOTS [32, 33]. Some TB-HIV patients are possible to have lack of insight towards their health seeking behaviour which leads towards loss to follow-up, including defaulting DOTS [34]. Other barriers are such as distance, transportation, and financial limitations, which require good collaboration with nongovernmental organizations, NGOs, such as by providing mileage allowance or providing transport to patients, resulting in better adherence to treatment and higher satisfaction towards the treatment [33].

By contrast, a qualitative study showed that the rigidity of DOTS was one of the factors of treatment nonadherence by patients with TB-HIV, which led to lost to follow-up and therefore unsuccessful treatment outcome [35].

Taking anti-TB medications for at least 6 months is another determinant factor for successful treatment of TB, which supports the present findings [36]. Most patients with TB-HIV are cured with a standard 6-month treatment regimen [12]. Another study comparing 6-month and 9-month treatment reported similar treatment outcomes but with significantly lower recurrence rates compared to a 6-month, thrice-weekly regimen [37]. It has also been proven that a longer treatment regimen can yield a more favourable treatment outcome for patients with TB-HIV [38], which supports our observations. Besides, low TB treatment adherence may lead to increased risk of drug resistance, treatment relapse, and mortality. A study found that patients with poor treatment compliance had two times shorter time to death as compared to those who comply to TB treatment [39]. Therefore, it is important for healthcare providers to ensure that patients with TB-HIV adhere to the TB treatment regimen [35].

The present study demonstrated that non-Malaysians had higher odds of having unsuccessful TB treatment outcome, but it was not statistically significant; thus, it was not included as a determinant in this study. This is in concordance with another study performed in Malaysia [26, 28]. The small number of non-Malaysian patients with TB-HIV could have contributed to the nonsignificant findings of both studies. Migration is a risk factor for TB, especially for migrants from high-TB burden countries. Immigrants tend to have a higher risk to lost to follow-up, which further contributes towards unsuccessful TB treatment outcome [36]. The WHO has emphasised efforts to control TB in order to assist governments worldwide in terms of policies for migrants by preventing HIV/AIDS among migrants, as they are a vulnerable group [40].

Here, lack of formal education, being unemployed, and low household income were significantly associated with unsuccessful treatment outcome, compared to having received formal education, being employed, and high household income. These findings are in concordance with other studies that show that people with low socioeconomic backgrounds tend to have a higher risk of poorer TB treatment outcome [26, 35, 36]. The risk of developing TB increases among people with low socioeconomic backgrounds, as they usually live in areas with poor ventilation, have poor knowledge and behavioural practices regarding the disease itself, and are malnourished, which may lead to low immunity [36].

This study found majority patients lived in medium/high cost areas and majority patients had low household income. Based on one's preference, housing aspects influenced people to make housing choices and what factors are important to them in considering where to live. Some of the aspects are such as near to the working place, amenities provided, and comfortable environment and surrounding. Thus, there could be possibility that respondents who have low household income willing to take risk in moving out to the higher rate of housing price probably exceed their affordability in order to fulfil their housing preference [41].

DM is a risk factor for developing TB. Similar to another study, the present findings show no significant difference in TB treatment outcome between patients with and without DM [28]. Patients with DM tend to have poorer TB treatment outcome compared to those without DM comorbidity, as DM patients with TB can have worsened glycaemic index [42]. However, patients with TB-HIV have low immunity due to the underlying HIV. On the other hand, patients with underlying HIV have higher chances of developing TB compared to patients with underlying DM [36].

The presence of a BCG scar may be a protective factor against developing TB infection. The present study suggests that patients with TB-HIV without a BCG scar have 4.2 times higher odds of having unsuccessful TB treatment outcome compared to patients with a BCG scar, but it was not significantly associated with unsuccessful TB treatment outcome. Likewise, this finding is supported by the findings of Nik Nor Ronaidi et al. [28].

Here, we found that not receiving ART was significantly associated with unsuccessful TB treatment outcome, and this is consistent with previous studies worldwide [8, 13, 14, 43, 44]. A study from Iran found that patients with TB-HIV who had not been started with ART prior had a higher chance of dying earlier. Physicians had limited time to start such patients on ART due to the shorter duration of hospitalisation because they died earlier [14]. Recent literature showed that the risk of death in patients with TB-HIV coinfection that did not receive ART were 3 times higher as compared to those who received ART [45]. Study shows that ART should be initiated as early as possible in MDR-TB patients with HIV coinfection [15]. It is important to start ART earlier among these patients, as higher mortality was found among TB-HIV patients who had not been started with ART prior [14]. It is also shown that higher risk for unsuccessful MDR-TB treatment occurs with the increase frequency of missed visits which were 1.50 times, 2.25 times, and 3.37 times for once missed visit, twice missed visit, and thrice missed visits, respectively [16].

In the present study, advanced CXR presentation was not significantly associated with unsuccessful TB treatment outcome. In contrast, advanced CXR findings have been suggested as a determinant factor for unsuccessful TB treatment outcome [28]. In patients with TB-HIV, up to 10–15% of such patients with proven TB may have normal CXR due to the delayed immune response [46].

Here, sputum smear upon diagnosis was not significantly associated with unsuccessful TB treatment outcome. The numbers of patients with smear-negative and smear-positive TB were almost identical in the present study, and this might explain why it was not associated with the treatment outcome. Nevertheless, this condition can also be due to the nonspecific symptoms and broad-spectrum immune response among patients with TB-HIV, which may produce false-negative sputum smear results among such patients [46–50]. These findings were concordant with that of Nguyen et al. and Nik Nor Ronaidi et al. [20, 28]. Others have, however, showed that positive-sputum smear is significantly associated with unsuccessful TB treatment outcome [1, 44].

Here, positive sputum culture upon diagnosis was significantly associated with unsuccessful TB treatment outcome. This finding was supported by similar findings by Prado et al., Nguyen et al., and Swaminathan et al. [1, 20, 37]. Patients with TB-HIV with positive sputum culture may have higher TB bacterial loads, which may thus worsen the prognosis. Sputum culture is more accurate for diagnosing TB and for determining the prognosis in patients with TB-HIV, eventhough their sputum smear is negative [48]. This is consistent, as sputum culture is the gold standard for TB diagnosis, especially among patients with HIV, as it has higher sensitivity compared to sputum smear [46, 51].

The present study identifies the determinants of TB-HIV treatment outcome, which could guide healthcare facilities, especially those in Kuala Lumpur, to focus on those areas for better treatment outcome among patients with TB-HIV, so that better treatment outcome can be achieved in the future. Other than that, the TB data were obtained from a reliable source (TBIS), which represents the population studied.

Nevertheless, this study has some limitations. Although the patients were selected randomly, they were all from the Kuala Lumpur Federal Territory Health Office registry. Hence, the outcome of this study is mainly limited to patients within the Federal Territory of Kuala Lumpur, and it is not known if it can be generalised to other states in Malaysia or to other countries. The data used in this study belong to the Ministry of Health of Malaysia and based on the ethical approval gained for this study, and only data for year 2013 till 2017 were approved to be used by Universiti Kebangsaan Malaysia. Second, as it was secondary data, it was very difficult to determine the sputum conversion rate after 2 months of treatment, as not all patients with TB-HIV have these data. Other than that, we could not assess the characteristic of the patient's immune status as the CD4 count are not available from the data we used. Besides, the transferred-out patients excluded from the study would produce bias results because they could not be included in the study due to the inability to assess the treatment outcome, as their records were unavailable.

## 5. Conclusions

Nearly 50% of patients with TB-HIV have unsuccessful TB treatment outcome. Crucial measures are needed to ensure that such patients receive DOTS and continuous TB treatment of >6 months. Healthcare settings are required to strengthen and widen DOTS service coverage and to prioritize DOTS, especially among the high-risk groups. Accordingly, rigorous follow-ups from healthcare professionals are needed to ensure intensified treatment adherence and better rates of successful TB treatment outcome among patients with TB-HIV.

## Figures and Tables

**Table 1:** Sociodemographic and socioeconomic characteristics of patients with TB-HIV coinfection (total patients, *n* = 235).

Sociodemographic characteristics	*n* (%)
Age (years)	
18–39	120 (51.1)
40–59	110 (46.8)
≥60	5 (2.1)

Sex	
Male	201 (85.5)
Female	34 (14.5)

Citizenship	
Malaysian	211 (89.8)
Non-Malaysian	24 (10.2)

Ethnicity	
Malay	115 (48.9)
Chinese	61 (26)
Indian	26 (11.1)
Others	33 (14)

Place of residence	
Low cost	64 (27.2)
Medium/high cost	146 (62.1)
Others (homeless/institutional)	25 (10.6)

Socioeconomic characteristics	
Formal education	
Yes	187 (79.6)
No	48 (20.4)

Employment status	
Employed	115 (48.9)
Unemployed	120 (51.1)

Household income (MYR)	
Low (<MYR3000)	208 (88.5)
High (≥MYR3000)	27 (11.5)

**Table 2:** Clinical characteristics of patients with TB-HIV coinfection (total patients, *n* = 235).

Clinical characteristics	*n* (%)
DM	
Yes	9 (3.8)
No	226 (96.2)

Smoking status	
Yes	114 (48.5)
No	121 (51.5)

BCG scar	
Present	213 (90.6)
Absent	22 (9.4)

ART	
Yes	92 (39.1)
No	143 (60.9)

Type of TB	
Pulmonary	174 (74)
Extrapulmonary	61 (26)

TB case category	
New case	199 (84.7)
Relapse	25 (10.6)
Return after lost to follow-up	11 (4.7)

CXR presentation upon diagnosis	
No/minimal lesion	159 (67.7)
Advanced	70 (29.8)
Not performed	6 (2.6)

Sputum smear upon diagnosis	
Positive	93 (39.6)
Negative	119 (50.6)
Not performed	23 (9.8)

Sputum culture upon diagnosis	
Positive	19 (8.1)
Negative	149 (63.4)
Not performed	67 (28.5)

DOTS status	
Yes	155 (66)
No	80 (34)

Duration of TB treatment (months)	
<6	103 (43.8)
6–12	113 (48.1)
≥12	19 (8.1)

TB treatment outcome	
Cured	47 (20)
Completed treatment	89 (37.9)
Died	72 (30.6)
Failure	22 (9.4)
Lost to follow-up	5 (2.1)

**Table 3:** Simple logistic regression (SLR) identification of factors associated with unsuccessful TB treatment outcome in patients with TB-HIV in Kuala Lumpur.

Variable	SLR					
	Unsuccessful outcome, *n* (%)	Successful outcome, *n* (%)	Unadjusted OR	95% CI	*χ* ^*2*^ (df)^a^	*P* value
Sociodemographic characteristics						
Age (years)					1.34 (2)	0.511
18–39	47 (39.2)	73 (60.8)	1			
40–59	49 (44.5)	61 (55.5)	1.25	0.74, 2.11	0.68 (1)^b^	0.409
≥60	3 (60)	2 (40)	2.33	0.38, 14.47	0.82 (1)^b^	0.364

Sex						
Male	82 (40.8)	119 (59.2)	0.69	0.33, 1.43	1.00 (1)	0.317
Female	17 (50)	17 (50)	1			

Citizenship						
Non-Malaysian	17 (70.8)	7 (29.2)	3.821	1.52, 9.61	9.00 (1)	0.003^*∗*^
Malaysian	82 (38.9)	129 (61.1)	1			

Ethnicity					11.67 (3)	0.009^*∗*^
Malay	44 (38.3)	71 (61.7)	0.31	0.14, 0.70	7.93 (1)^b^	0.005^*∗*^
Chinese	20 (32.8)	41 (67.2)	0.24	0.10, 0.60	9.45 (1)^b^	0.002^*∗*^
Indian	13 (50)	13 (50)	0.50	0.17, 1.44	1.66 (1)^b^	0.198
Others	22 (66.7)	11 (33.3)	1			0.813

Place of residence					0.41 (2)	
Others (homeless/institutional)	12 (48)	13 (52)	1.32	0.57, 3.10	0.42 (1)^b^	0.519
Low cost	27 (42.2)	37 (57.8)	1.05	0.58, 1.90	0.02 (1)^b^	0.882
Medium/high cost	60 (41.1)	86 (58.9)	1			

Socioeconomic characteristics						
Formal education						
No	31 (64.6)	17 (87.5)	3.19	1.65, 6.19	12.38 (1)	<0.001^*∗*^
Yes	68 (36.4)	119 (63.6)	1			

Employment status						
Unemployed	60 (50)	60 (50)	1.95	1.15, 3.30	6.27 (1)	0.012^*∗*^
Employed	39 (33.9)	76 (66.1)	1			

Household income						
Low	93 (44.7)	115 (55.3)	2.83	1.10, 7.30	5.31 (1)	0.021^*∗*^
High	6 (22.2)	21 (77.8)	1			

Clinical characteristics						
DM						
Yes	3 (33.3)	6 (66.7)	0.68	0.17, 2.78	0.30 (1)	0.581
No	96 (42.5)	130 (57.5)	1			

Smoking status						
Yes	49 (43)	65 (57)	1.07	0.64, 1.80	0.07 (1)	0.797
No	50 (41.3)	71 (58.7)	1			

BCG scar						
Absent	16 (72.7)	6 (27.3)	4.18	1.57, 11.11	9.32 (1)	0.002^*∗*^
Present	83 (39)	130 (61)	1			

ART						
Yes	30 (32.6)	62 (67.4)	1.93	1.12, 3.33	5.69 (1)	0.017^*∗*^
No	69 (48.3)	74 (51.7)	1			

Type of TB						
Pulmonary	73 (42)	101 (58)	0.97	0.54, 1.76	0.01 (1)	0.927
Extrapulmonary	26 (42.6)	35 (57.4)	1			

TB case category					0.19 (2)	0.910
Relapse	11 (44)	14 (56)	1.08	0.47, 2.49	0.03 (1)^b^	1.076
Return after lost to follow-up	4 (36.4)	7 (63.6)	0.78	0.22, 2.76	0.15 (1)^b^	0.782
New case	84 (42.2)	115 (57.8)	1			

** **CXR presentation upon diagnosis					7.75 (2)	0.021
Not performed	5 (83.3)	1 (16.7)	1.70	0.96, 2.99	3.31 (1)^b^	0.069
Advanced	35 (50)	35 (50)	8.48	0.97, 74.30	3.72 (1)^b^	0.054
No/minimal lesion	59 (37.1)	100 (62.9)	1			

Sputum smear upon diagnosis					0.12 (2)	0.944
Positive	40 (43)	53 (57)	1.04	0.60, 1.80	0.02 (1)^b^	0.884
Not performed	9 (39.1)	14 (60.9)	0.89	0.36, 2.21	0.07 (1)^b^	0.797
Negative	50 (42)	69 (58)	1			

Sputum culture upon diagnosis					15.14 (2)	0.001
Positive	14 (73.7)	5 (26.3)	5.54	1.89, 16.26	9.73 (1)^b^	0.002^*∗*^
Not performed	35 (52.2)	32 (47.8)	2.17	1.20, 3.90	6.64 (1)^b^	0.010^*∗*^
Negative	50 (33.6)	99 (66.4)	1			

DOTS status						
No	71 (88.8)	9 (11.3)	35.78	15.99, 80.05	117.22 (1)	<0.001^*∗*^
Yes	28 (18.1)	127 (81.9)	1			

Duration of TB treatment (months)					206.98 (2)	<0.001^*∗*^
<6	93 (90.3)	10 (9.7)	79.05	15.90, 393.03	28.52 (1)^b^	<0.001^*∗*^
6–12	4 (3.5)	109 (96.5)	0.31	0.05, 1.84	1.66 (1)^b^	0.198
≥12	2 (10.5)	17 (89.5)	1			

^a^Likelihood ratio (LR) test. ^b^Wald test. ^*∗*^Significant at *p* < 0.05. CI, confidence interval. *d*f, degree of freedom.

**Table 4:** Significant determinant factors of TB treatment outcomes in patients with TB-HIV in Kuala Lumpur, the final model (total patients, *n* = 235).

Characteristic	SLR				MLR			
	Unadjusted OR	95% CI	*χ* ^*2*^ (df)^a^	*P* value	aOR^c^	95% CI	*χ* ^*2*^ (df)^a^	*P* value
DOTS status								
No	35.78	15.99, 80.05	117.221 (1)	<0.001^*∗*^	21.71	5.36, 87.94	24.52 (1)	<0.001^*∗*^
Yes	1	—	—	—	1	—	—	—

Duration of TB treatment (months)			206.98 (2)	<0.001^*∗*^				
<6	79.05	15.90, 393.03	28.52 (1)^b^	<0.001^*∗*^	34.54	5.97, 199.93	15.63 (1)^b^	<0.001^*∗*^
6–12	0.31	0.05, 1.84	1.66 (1)^b^	0.198	0.19	0.03, 1.38	2.72 (1)^b^	0.099
≥12	1	—	—	—	1	—	—	—

^a^Likelihood ratio (LR) test. ^b^Wald test. ^c^Adjusted for citizenship status, ethnicity, formal education, employment status, household income, BCG scar, ART, CXR upon diagnosis, sputum culture upon diagnosis, DOTS status, and duration of TB treatment using the forward LR method. ^*∗*^*p* < 0.05. There was no multicollinearity (VIF <10) and no interaction problem. 1, reference; SLR, simple logistic regression; MLR, multiple logistic regression; OR, odds ratio; aOR, adjusted odds ratio; d*f,* degree of freedom.

## Data Availability

The datasets generated and analysed in this study are not publicly available due to restriction as they contain health information that could compromise the privacy of research participants but are available from the corresponding author upon request.
